# Optimizing the Dose of Pre-Pandemic Influenza Vaccines to Reduce the Infection Attack Rate

**DOI:** 10.1371/journal.pmed.0040218

**Published:** 2007-06-19

**Authors:** Steven Riley, Joseph T Wu, Gabriel M Leung

**Affiliations:** Department of Community Medicine and School of Public Health, Li Ka Shing Faculty of Medicine, The University of Hong Kong, Hong Kong Special Administrative Region, China; Harvard School of Public Health, United States of America

## Abstract

**Background:**

The recent spread of avian influenza in wild birds and poultry may be a precursor to the emergence of a 1918-like human pandemic. Therefore, stockpiles of human pre-pandemic vaccine (targeted at avian strains) are being considered. For many countries, the principal constraint for these vaccine stockpiles will be the total mass of antigen maintained. We tested the hypothesis that lower individual doses (i.e., less than the recommended dose for maximum protection) may provide substantial extra community-level benefits because they would permit wider vaccine coverage for a given total size of antigen stockpile.

**Methods and Findings:**

We used a mathematical model to predict infection attack rates under different policies. The model incorporated both an individual's response to vaccination at different doses and the process of person-to-person transmission of pandemic influenza. We found that substantial reductions in the attack rate are likely if vaccines are given to more people at lower doses. These results are applicable to all three vaccine candidates for which data are available. As a guide to the magnitude of the effect, we simulated epidemics based on historical studies of immunogenicity. For example, for one of the vaccines for which data are available, the attack rate would drop from 67.6% to 58.7% if 160 out of the total US population of 300 million were given an optimal dose rather than 20 out of 300 million given the maximally protective dose (as promulgated in the US National Pandemic Preparedness Plan). Our results are conservative with respect to a number of alternative assumptions about the precise nature of vaccine protection. We also considered a model variant that includes a single high-risk subgroup representing children. For smaller stockpile sizes that allow vaccine to be offered only to the high-risk group at the optimal dose, the predicted benefits of using the homogenous model formed a lower bound in the presence of a risk group, even when the high-risk group was twice as infective and twice as susceptible.

**Conclusions:**

In addition to individual-level protection (i.e., vaccine efficacy), the population-level implications of pre-pandemic vaccine programs should be considered when deciding on stockpile size and dose. Our results suggest that a lower vaccine dose may be justified in order to increase population coverage, thereby reducing the infection attack rate overall.

## Abstract 2

Steven Riley and colleagues examine the potential benefits of "stretching" a limited supply of vaccine and suggest that substantial reductions in the attack rate are possible if vaccines are given to more people at lower doses.

## Editors' Summary

**Background.:**

Every winter, millions of people catch influenza, a viral infection of the nose, throat, and airways. Most recover quickly, but the disease can be deadly. In the US, seasonal influenza outbreaks (epidemics) cause 36,000 excess deaths annually. And now there are fears that an avian (bird) influenza virus might trigger a human influenza pandemic—a global epidemic that could kill millions. Seasonal epidemics occur because flu viruses continually make small changes to their hemagglutinin and neuraminidase molecules, the viral proteins (antigens) that the immune system recognizes. Because of this “antigenic drift,” an immune system response (which can be induced by catching flu or by vaccination with disabled circulating influenza strains) that combats flu one year may provide only partial protection the next year. “Antigenic shift” (large changes in flu antigens) can cause pandemics because communities have no immunity to the changed virus.

**Why Was This Study Done?:**

Although avian influenza virus, which contains a hemagglutinin type that differs from currently circulating human flu viruses, has caused a few cases of human influenza, it has not started a human pandemic yet because it cannot move easily between people. If it acquires this property, which will probably involve further small antigenic changes, it could kill millions of people before scientists can develop an effective vaccine against it. To provide some interim protection, many countries are preparing stockpiles of “pre-pandemic” vaccines targeted against the avian virus. The US, for example, plans to store enough pre-pandemic vaccine to provide maximum protection to 20 million people (including key health workers) out of its population of 300 million. But, given a limited stockpile of pre-pandemic vaccine, might giving more people a lower dose of vaccine, which might reduce the number of people susceptible to infection and induce herd immunity by preventing efficient transmission of the flu virus, be a better way to limit the spread of pandemic influenza? In this study, the researchers have used mathematical modeling to investigate this question.

**What Did the Researchers Do and Find?:**

To predict the infection rates associated with different vaccination policies, the researchers developed a mathematical model that incorporates data on human immune responses induced with three experimental vaccines against the avian virus and historical data on the person–person transmission of previous pandemic influenza viruses. For all the vaccines, the model predicts that giving more people a low dose of the vaccine would limit the spread of influenza better than giving fewer people the high dose needed for full individual protection. For example, the researchers estimate that dividing the planned US stockpile of one experimental vaccine equally between 160 million people instead of giving it at the fully protective dose to 20 million people might avert about 27 million influenza cases in less than year. However, giving the maximally protective dose to the 9 million US health-care workers and using the remaining vaccine at a lower dose to optimize protection within the general population might avert only 14 million infections.

**What Do These Findings Mean?:**

These findings suggest that, given a limited stockpile of pre-pandemic vaccine, increasing the population coverage of vaccination by using low doses of vaccine might reduce the overall influenza infection rate more effectively than vaccinating fewer people with fully protective doses of vaccine. However, because the researchers' model includes many assumptions, it can only give an indication of how different strategies might perform, not firm numbers for how many influenza cases each strategy is likely to avert. Before public-health officials use this or a similar model to help them decide the best way to use pre-pandemic vaccines to control a human influenza pandemic, they will need more information about the efficacy of these vaccines and about transmission rates of currently circulating viruses. They will also need to know whether pre-pandemic vaccines actually provide good protection against the pandemic virus, as assumed in this study, before they can recommend mass immunization with low doses of pre-pandemic vaccine, selective vaccination with high doses, or a mixed strategy.

**Additional Information.:**

Please access these Web sites via the online version of this summary at http://dx.doi.org/10.1371/journal.pmed.0040218.

- US Centers for Disease Control and Prevention provide information on influenza and influenza vaccination for patients and health professionals (in English, Spanish, Filipino, Chinese, and Vietnamese)
- The World Health Organization has a fact sheet on influenza and on the global response to avian influenza (in English, Spanish, French, Russian, Arabic, and Chinese)
- The MedlinePlus online encyclopedia devotes a page to flu (in English and Spanish)
- The UK Health Protection Agency information on avian, pandemic, and seasonal influenza
- The US National Institute of Allergy and Infectious Diseases has a comprehensive feature called “focus on the flu”

## Introduction

The recent spread of H5N1 highly pathogenic avian influenza (HPAI) in wild birds and poultry may be a precursor to the emergence of a 1918-like human pandemic [1,2]. Therefore, stockpiles of human pre-pandemic vaccine (targeted at avian HPAI strains) are being considered by many countries. For example, the US intends to provide enough pre-pandemic vaccine to protect 20 million people [3]. Data from Phase II clinical trials are available for three candidate vaccines [4–6]. Two of the candidates are adjuvant inactivated whole-virion vaccines for which immunological responses for doses in the ranges 1.25–10 μg [5] and 7.5–30 μg [4] have been reported. The third candidate is a nonadjuvant inactivated split-virion vaccine [6] for which immunological responses for doses in the range 7.5–90 μg have been reported. All three trials found that during haemagglutinin inhibition (HI) tests, sera from at least 50% of individuals who received two inoculations at the maximum dose were able to neutralize target antigens at concentrations of 1:40. It should be noted that pre-pandemic vaccine stockpiles would most likely be used as part of a globally reactive strategy; i.e., countries would plan to initiate vaccination programs when a nascent pandemic is confirmed in a remote region, rather than routinely vaccinating against avian strains. However, it would not be possible to use vaccines in a truly reactive way, i.e., vaccinating contacts of individual cases, because the time lag between vaccination and protection is long compared with the likely speed of progression of individual national epidemics.

The current annual global production capacity is 350 million doses of trivalent influenza vaccine [7]. If pre-pandemic vaccine stockpiles are implemented, they will need to be replenished periodically because of strain drift in HPAI [8]. In addition, capacity will need to be shared with vaccines against seasonal influenza. Therefore, even if global capacity is increased substantially, say to 780 million doses by 2009 as proposed by the World Health Organization [7], it remains unlikely that sufficient pre-pandemic vaccine antigen will ever be available for many populations to allow universal coverage at the maximally protective doses reported for current candidates.

This excess demand for pandemic vaccination has stimulated a vigorous debate over appropriate goals for vaccine allocation strategies. Some have argued that the distribution of vaccines should be designed to directly protect those most at risk of mortality or severe morbidity [9], while others have indicated that vaccination of those groups who are most infectious should be prioritized as this will have substantial indirect effects [10]. A recent comparison [11] of these two approaches suggests that the latter (transmission-limiting strategies) would be more successful for moderate- or low-transmission pandemic strains, whereas the former (morbidity-limiting strategies) would be more effective for higher-transmissibility strains.

Here, we investigate a parallel issue that policy makers should consider before deciding on pandemic vaccine allocation strategies. We suggest that for many large populations, the principal constraint for pre-pandemic influenza vaccine stockpiles will be the total mass of antigen maintained. In this article, we predict the pandemic influenza attack rate for different dosing strategies under this constraint using a mathematical model that incorporates both an individual's response to vaccination and the process of person-to-person transmission. We investigate the hypothesis that lower individual doses, conferring less than maximal protection for those vaccinated, may provide substantial incremental community-level benefit because they would permit wider vaccine coverage for a given size of antigen stockpile.

## Methods

### Simple Illustrative Model

The main concepts of our approach can best be understood using a simplified model of immune response. For this simple model, we assumed that the action of an influenza vaccine was all-or-nothing [12], i.e., it conferred complete protection on a proportion *p_v_* of those who received it but gave no protection to the remaining 1 − *p_v_*. We defined *c* as the proportion of the population that received the vaccine and *f(d)* to be the dose-response function, such that *p_v_* = *f(x)* was the probability of complete protection after vaccination where dose *x* was one of the doses tested during the clinical trial. Because the only action of this vaccination program was to completely protect a proportion *cp_v_* of the population, it must have been optimally effective when *cp_v_* was maximized. It is straightforward to show that this occurs when *f(x)*/*x* is maximized. Note that this optimality condition is independent of transmissibility of the pathogen. The function *f(x)*/*x* is the gradient of a straight line from the origin to a point on the dose-response curve *y = f(x)*.

The basic reproductive number *R*_0_ is a measure of the transmissibility of a pathogen and is defined as the average number of infections generated by a typically infectious individual in an otherwise susceptible population. We assumed mass-action-like mixing and that all individuals had similar infectiousness profiles. Therefore, for this simple model of vaccine effect, the infection attack rate *a* was defined by a single characteristic equation for the product of the initial proportion susceptible and the overall probability of infection,

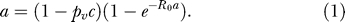



This equation predicts that in the absence of vaccination, for a pandemic strain with *R*_0_ *=* 1.8, the infection attack rate *a =* 73%. After two doses with 10 μg of the vaccine described by Lin et al. [6], 78% of HI titres reached 1:40 or greater. Conservatively, if we assume that 50% of individuals with HI titres of 1:40 or greater are protected [13], this implies that 39% of individuals were protected. Similarly, after two doses with 2.5 μg—at which *f(x)*/*x* is maximized – 14.5% of individuals receiving the vaccine were protected. Therefore, under this simple model, if 20 out of 300 million people in the US were vaccinated with two 10 μg doses, 20/300 × 39% = 2.6% of the population would be completely protected and the attack rate *a* would drop from 73.2% to 69.5% for *R*_0_ *=* 1.8,whereas if the same total amount of antigen were used to vaccinate 80 out of 300 million with two 2.5 μg doses, 80/300 × 14.5% = 3.8% of the population would be completely protected and the attack rate *a* would drop to 67.7% (note that these numerical examples are for illustrative purposes only).

### Model Used for Quantitative Results

However, results from this simple model substantially underestimate the community-level effect of partial coverage vaccination programs, because the model does not capture the full range of possible immune responses. In order to more accurately assess the impact of vaccination, we refined the simple model to include a continuous range of possible vaccine doses and multiple immune states for post-vaccination individuals. For each candidate vaccine we defined: the ordered set of all doses tested during the clinical trial, **x** = (*x_1_,…,x_j_,…,x_m_*}; the continuous dose *d* between the lowest *(x_1_)* and highest *(x_m_)* doses tested; and the set of all immune classes (defined by HI titre) used in the clinical trial, **h** = (*h_1_,…,h_j_,…,h_n_*}. The probability mass *p_T_(h_i_;x_j_)* was the probability that vaccination with one of the trial doses *x_j_* induced immune state *h_i_*. Values for *p_T_(h_i_;x_j_)* were assumed to be equal to the proportion of trial participants observed in each immune class. Similarly, the probability mass *p(h_i_;d)* was defined as the probability that vaccination with dose *d* (drawn from a continuous scale) induced immune state *h_i_*. Values for *p(h_i_;d)* were obtained by logarithmic interpolation in the *d* dimension between the largest member of **x** smaller than *d* and the smallest member of **x** larger than *d*.

The susceptibility of all those in *h_i_*, the *i*th immune state, was reduced by a factor *z_i_* (i.e., *z_i_* = 0 was fully susceptible) according to the attack rates observed during deliberate infection experiments with the post-1968 HK strain of H3N2 influenza A (Table S1) [14]. Individuals were assumed to have been infected if there was a 4-fold or greater increase in serum HI titre 2 or 3 wk after the challenge, or if virus could be cultured from nasal swabs taken 48 h after the infectious challenge. For each of these experiments, we used the class with the highest attack rate as the baseline for susceptibility, effectively imposing the constraint that the relation between HI titre and protection is a monotonically decreasing function. Therefore, for the pre-1968 strain [14], we used the infection rate of the 1:6 group as the baseline for susceptibility. Although, qualitatively, our results are not sensitive to this assumption, it does affect the relative protective effect of the lower antibody classes and therefore reduces the estimated benefits of lower-dose policies (Figure S1; Table S1). The relative susceptibility of the *i*th class was equal to the attack rate observed in that class divided by that observed in the baseline class. For example, for the pre-1968 strain, 41.9% of volunteers in the fourth HI titre class (1:24) were infected during the experiment, compared with 74.3% of volunteers in the 1:6 HI titre class. Therefore, *z*_4_ = 1 − 41.9/74.3 = 0.44. Because different HI titre classes were used for each vaccine trial and for the deliberate infection experiment, we used logarithmic interpolation to generate a continuous function for *z_i_* values.

**Figure 1:**
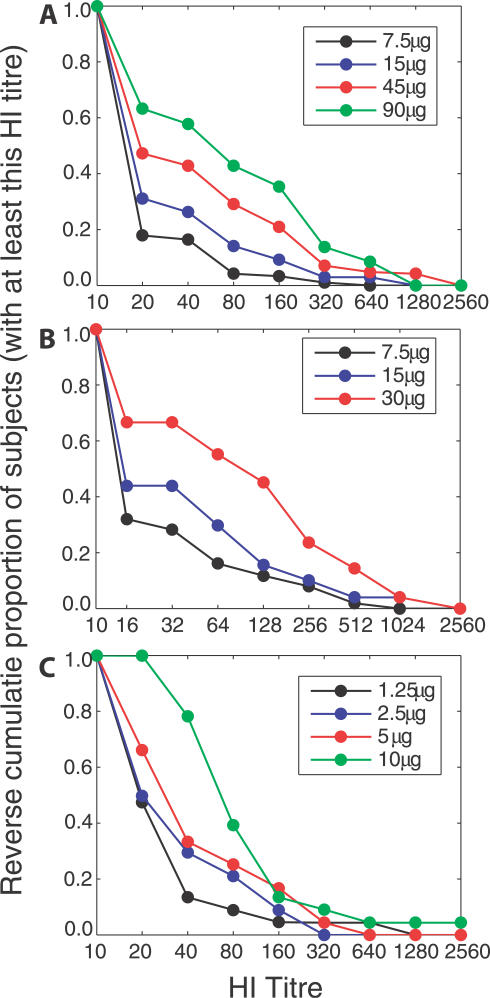
Individual-Level Vaccine Response for Three Vaccine Candidates Vaccine candidates were those reported by (A) Treanor et al. [6] (on day 56 after the initial dose), (B) Bresson et al. [4] (day 42, with alum) and (C) Lin et al. [5] (day 42). The legends show the range of doses included in the clinical trial for each vaccine. The y-axis shows the reverse cumulative proportion of participants with HI titres (x-axis). Each titre level constitutes an immune state *h_i_*. We assume that *p_T_(h_i_;x_j_)* is equal to the logarithmic interpolation between the discrete doses (see main text).

By assuming that all those in the same immune state had their susceptibility reduced by a factor *z_i_*, immunity in this version of the model could be described as “leaky” (as opposed to all-or-nothing [12]), because no individual was fully protected. We also used a model variant in which a proportion *z_i_* of those in the *i*th immune class were assumed to be completely protected while the rest were not (this variant is referred to below as “all-or-nothing within immune classes”). We define *p(z_i_;d)* as the probability that vaccination at dose *d* puts individuals into an immune state with average reduction in susceptibility *z_i_*. The function *p(z_i_;d)* is shown graphically for the three candidate vaccines in Figure 1. We were also interested in the expected reduction in susceptibility for a given dose, which we define to be

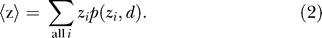



This function summarizes the combined effect of a vaccine altering the immune state of an individual and of the protective effects of different immune states.

We then calculated the infection attack rate under this refined individual model in a population of well-mixed risk groups. We let *x_ij_* be the proportion of a population in immune state *i* and risk group *j* after a vaccination program with coverage *c_j_* in risk group *j*. The first immune class of a given risk group, in which there was no protection against the pandemic strain, contained those who had not received vaccination and those for which it had had no effect *x_1j_* = 1 − *c_j_* + *c_j_* *p*. The proportion of the overall population in the other immune states, *i* > 1, was *x_ij_ = c_j_p_i_*. We considered only scenarios with two risk groups, one high and one low. The high-risk group was 1 + *α* times as infectious and 1 + *ɛ* times as susceptible, but the two groups mixed freely. Therefore, the infection matrix between these risk groups was

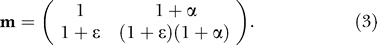



Thus, our equation for the infection attack rate could be modified for each risk group *j* to include contributions from individuals in each of the different post-vaccination immune states in each of the two risk groups,

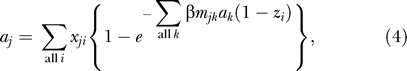
where *a_j_* was the infection attack rate of the *j*th risk group and *m_jk_* was an element of **m**. In the homogeneous case, with only a single risk group, *α* = 0, *ɛ* = 0, *m*_*ij*_ = 1; *β* was equal to the basic reproductive number for the model. In the heterogeneous case, with at least one of *α* > 0 or *ɛ* > 0, the value of *β* was chosen to give an overall attack rate of 73% in the absence of vaccination (the attack rate in the homogeneous case when *R*_0_ = 1.8).

The structure described above constitutes our base case model. We tested the sensitivity of our results with the homogeneous model to assumptions concerning the reference data for the protection associated with immune states (i.e., 1968 HK strain of H3N2 influenza A [14]) and to the assumption of leaky versus all-or-nothing immunity within immune states. Note that we use infection as our outcome measure for this study; we do not address the relationship between infection and either morbidity or mortality.

## Results

**Figure 2:**
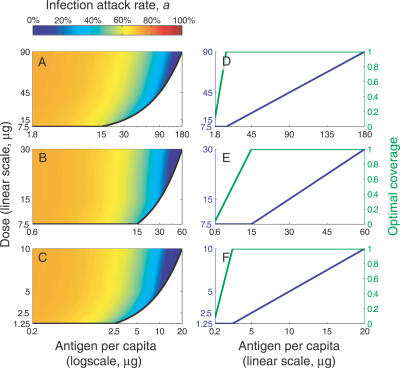
Impact of Alternative Dosing Strategies for the Three Vaccines Vaccines candidates were those reported by (A, D) Treanor et al. [6]; (B, E) Bresson et al. [4]; and (C, F) Lin et al. [5]. (A–C) Infection attack rate *a* (colour as per legend) for different dosing strategies (y-axes) and different stockpile sizes (antigen per capita, x-axes). The solid black line shows the optimal dosing strategy at which the infection attack rate is minimized. Note that where the thick black line is not horizontal; it corresponds to (antigen per capita) = 2 × (dose). This is because each individual requires two doses of vaccine. (D–F) Illustration of the relationship between optimal coverage (proportion of population receiving vaccine, green lines, right y-axes) and optimal dose (blue lines, left y-axes) for different stockpile sizes (antigen per capita, x-axes).

Initially, we considered a homogeneous population without different risk groups. For all three vaccine candidates [4–6], increasing the population coverage by lowering the dose led to substantially lower infection attack rates (see Figure 2A–2C). However, the specific shape of the response curves for the different vaccines (Figure 1) influenced the expected degree of reduction for a given change in dose. For example, halving the dose from the maximum (therefore doubling the coverage) had a large impact on the infection attack rate for the vaccine reported by Treanor et al. [6] (hereafter, the Treanor vaccine), a less substantial effect for the vaccine reported by Lin et al. [5] (Lin vaccine), and very little change for the vaccine reported by Bresson et al. [4] (Bresson vaccine). If the optimal dose gave an attack rate within 1% of that of the minimum dose, we set the optimal dose to be the minimum. Under this criterion, for stockpile sizes too small to provide a minimum dose for all, the optimal dose for all three vaccine candidates (black lines, Figure 2A–2C) was equal to the minimum dose tested. For these small stockpiles, optimal coverage was less than 100% (green lines, Figure 2D–2F). For larger stockpile sizes, the optimal dose corresponded to an equal division of antigen among all members of the population.

We tested the sensitivity of our results to alternative assumptions about the protective effect of different HI titres, to the choice between leaky or all-or-nothing response types within an immune state and to the possible reservation of a portion of the stockpile to provide maximum individual protection to health care workers (Table 1). For illustrative purposes, we described these sensitivities using the predicted difference in attack rate if the optimal dose was used rather than the maximally protective dose, for stockpiles of antigen sufficient to vaccinate 20 out of 300 million Americans with the maximally protective dose (see above and [3]). For the Lin vaccine, under the baseline model the predicted attack rate dropped from 67.6% to 58.7%, giving an absolute reduction in attack rate of 8.9%. This relatively large reduction, compared with a drop of 1.8% under the simple model (see Methods), demonstrates the importance of including more realistic assumptions about the nature of individual immune responses to vaccination. When we recalibrated the baseline model using deliberate infection data from a second H3 strain [14] and field data [15], even larger drops in the attack rate were observed. Similarly, if we assumed an all-or-nothing response type within immune states, the benefits associated with an optimal dose also increased. In this sense, the results presented in Figure 2 are conservative with respect to alternative assumptions about the protective effect of different HI titres and to our choice between leaky or all-or-nothing response types within an immune state.

**Table 1:**
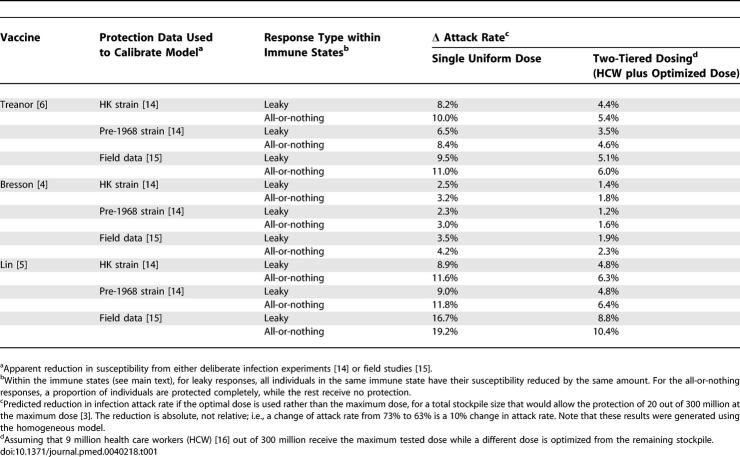
Sensitivity Analyses

Next, we assumed that the maximum dose was offered to 9 million health care workers (out of a population of 300 million in the United States [16]), while a different dose was optimized from the remaining stockpile in order to reduce the attack rate (Table 1). We assumed that health care workers were equally as infectious and susceptible as the rest of the population (see Discussion). As might be expected, the allocation of 45% of the stockpile to an inefficient maximum dose led to a reduction approximately by a factor of 2 in the estimated benefits of the overall vaccination program. Note that we focus on the US because it is the first country to publicly commit to a stockpile that will not be nationally universal. In contrast, Switzerland has committed to purchasing 8 million doses of pre-pandemic vaccine, which will be sufficient to cover its entire population [17].

**Figure 3:**
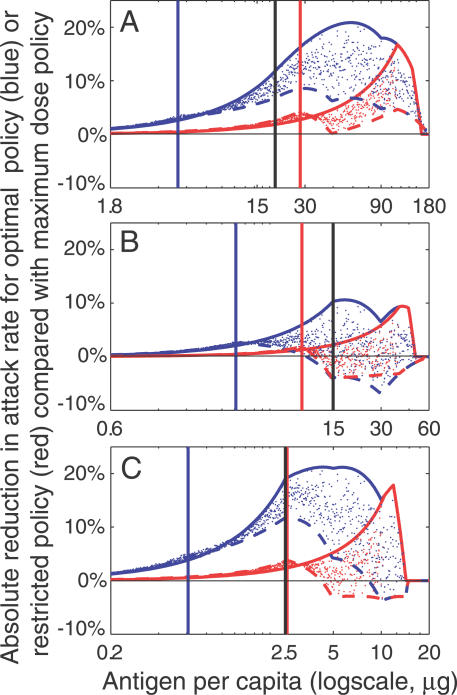
Sensitivity of Predicted Reduction in Attack Rate to the Inclusion of a High-Risk Group Representing Children for Three Vaccine Candidates (A) Treanor et al. [6]. (B) Bresson et al. [4]. (C) Lin et al. [5]. The underlying level of transmission *β* was calculated so as to maintain the same attack rate as the homogeneous model with *R*_0_ = 1.8, i.e., 73%. The blue lines and dots on the chart describe results for optimal policies, with doses at or very close to the minimum tested range, as calculated using the homogeneous model. The red lines and dots describe results for a restricted policy, i.e., the dose used had to be large enough to provide an expected reduction in the susceptibility of an individual of 〈*z*〉 = 0.4 (see Figure 4). Solid lines (not perfectly vertical) show the predicted reduction in infection attack rate for the homogeneous model, i.e., relative infectivity α = 0 and relative susceptibility *ɛ* = 0. The dashed lines show the benefit of the two different policies with relative infectivity of the risk group twice that of the rest of the population, i.e., *α* = 1, and relative susceptibility twice that of the rest of the population, i.e., *ɛ* = 1. Dots show the results from 1,000 Latin-hypercube samples [27] over the linear range for *α* and *ɛ,* and the log range of the x-axes for stockpile size. The red and blue vertical lines show the stockpile size at which complete coverage of the high-risk group is first achieved. The vertical black line shows the smallest stockpile size at which the whole population can be offered the optimal dose.

Up to this point, we assumed that all individuals were equally susceptible and infectious. However, if it is possible to target vaccination programs on the basis of transmission risk groups, as would be the case for a children-first policy, then the correlation between population subgroup and intervention must be reflected in the model structure. Therefore, in order to consider the impact of the optimal vaccination policies in heterogeneous populations, we returned to the baseline model described above (leaky immunity, conservative relationship between HI titre and protection, and no special provision for health care workers) but allowed a single high-risk subgroup of the population to be more susceptible or more infectious or both. This subgroup represented children and was set to be 24.5% of the population, which is the current proportion of the US population under 18 y of age [18]. Children were given priority when there was insufficient vaccine to cover the whole population, and were assumed to have a relative susceptibility of (1 + *α*) and a relative infectivity of (1 + *ɛ*). Figure 3 (blue dots) shows the performance of the optimal policy (calculated assuming that the population was homogeneous, i.e., as shown in Figure 2) for all three vaccines, for different stockpile sizes, with both *α* and *ɛ* allowed to vary randomly between 0 and 1. This model structure was equivalent to assuming that children could be, on average, up to twice as infectious and twice as susceptible which is consistent with recent estimates [19].

**Figure 4:**
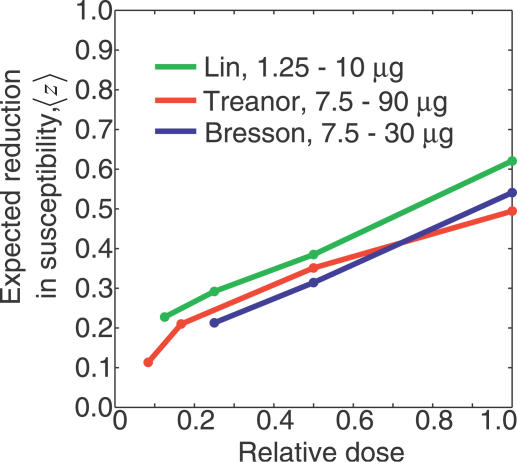
Expected Reduction in Susceptibility after Vaccination 〈*z*〉 for the Three Vaccines Vaccine candidates were those reported by Treanor et al. [6] (red); Bresson et al. [4] (blue); and Lin et al. [5] (green). For comparison, the dose (x-axis) is given as a proportion of the maximum dose used in the clinical trial. The range of doses used for each vaccine is indicated in the legend.

The inclusion of a high-risk group had different effects for stockpiles of different sizes. For smaller stockpiles, when the optimal dose could only be offered within the high-risk group, the presence of the high-risk group increased the benefits of using the optimal dose instead of the maximum dose. This might have been expected, given that we kept the attack rate constant after adding the high-risk group, thus forcing a higher proportion of infections to originate from individuals in the high-risk group than in the rest of the population. Therefore, preventing a given number of infections in the high-risk group (when one existed) had more of an effect than preventing the same number of infections in a homogeneous population.

For larger stockpile sizes, the presence of the high-risk group tended to reduce the benefits associated with the use of an optimal dose compared with a maximum dose. For the Treanor vaccine and for all but the largest stockpile sizes for the Lin vaccine, the lower bounds of those decreased benefits were still substantial improvements over the maximum-dose strategy for moderate stockpile sizes. However, for the Bresson vaccine, for stockpiles large enough to offer the minimum dose to the entire population, a substantial proportion of the parameter sets tested in our sensitivity analysis resulted in higher attack rates for the optimal dose (calculated using the homogeneous model) over the maximum dose.

In practice, there may be a lower bound on the level of individual protection that people will accept from a vaccine, even during a pandemic. Individual protective effects of different doses of candidate vaccines can be summarized by 〈*z*〉, the expected reduction in susceptibility (see Figure 4). Values for 〈*z*〉 varied between 0.1 and 0.6 across different doses for the three candidate vaccines. This quantity describes the anticipated level of individual protection per infectious challenge for those who receive vaccinations. It is of the same dimension as parameters used elsewhere to describe the reduction in susceptibility for individuals receiving prophylactic antiviral drugs (e.g., *θ* or 1/*AVE_S_* in [20]), but it is not a measure of the reduced risk of infection over a typical inter-pandemic influenza season: it does not specify how individuals respond to multiple infectious challenges. Any decision to increase coverage by reducing the vaccine dose implies a reduction in 〈*z*〉, with large reductions potentially unacceptable to vaccinees. With this potential restriction on vaccination policies in mind, we repeated the risk group analyses for all three candidate vaccines with a lower bound of 〈*z*〉 = 0.4 (red dots, Figure 3). Because the marginal return in protection for all three vaccines is highest at low doses, the imposition of this lower bound for an individual level of protection substantially reduced the community-level benefits of vaccination.

## Discussion

For a stockpile of pandemic influenza vaccine that is constrained by total mass of antigen, we have shown that for the three candidates for which data are available [4–6], wider coverage at lower doses would likely result in substantially lower infection attack rates. A reduction in attack rate of 8.9% (see Table 1) in a population the size of the US (300 million) corresponds to 27 million fewer influenza infections in a period of less than a year. Even if 45% of the stockpile were reserved to be used at maximum dose for health care workers, a reduction in attack rate of 4.8% implies that 14 million infections could be averted. Although these results are sensitive to some uncertainties (see below), the general pattern of lower doses being substantially more efficient at the population level is consistent across all three vaccines candidates and over a number of alternatives for key assumptions (Table 1).

By its very nature, a stockpile of pre-pandemic vaccine that is manufactured prior to the widespread transmission of a novel influenza strain represents a substantial gamble on the part of health policy makers. Therefore, in light of our results, we suggest that countries currently planning such programs immediately investigate the manufacturing and logistical implications of lower-dose, higher-coverage programs. If these downstream logistical constraints can be overcome, lower-dose vaccination programs will help to spread the risk of this resource-intensive public health policy by increasing the number of individuals who may benefit while reducing the expected infection attack rate. However, further empirical studies are required before final decisions with respect to pre-pandemic vaccines in general are made.

The results presented here depend two key assumptions. The first is that a logarithmic interpolation of clinical trial data at a few discrete doses gives an accurate description of the biological effect of vaccines across a continuous range of doses. This biological effect is measured by the expected distribution of homologous HI titres in naïve individuals following vaccination with a given dose. Data from clinical trials with greater resolution at low doses could rapidly address this uncertainty.

The second key assumption is more problematic and has implications beyond the planning of pre-pandemic vaccination programs. For our baseline results, we have assumed that the individual protection implied by the homologous avian H5 titre against the eventual pandemic strain is the same as that implied by the homologous human H3 titres of 345 volunteers who subsequently received a single experimental infectious challenge with a strain containing the same H3 protein [14]. We used these data for our baseline because of the large sample size and because the resulting estimates of reduction in attack rate were conservative compared with the use of alternative datasets (see Table 1). Also, these data appear to be the main source for the widely quoted HI titre protection threshold of 1:40. However, this apparently frail assumption (which implicitly motivates many empirical studies of influenza vaccines; see also Figure S1) highlights the urgent need for deliberate infection studies using currently circulating human influenza strains and current vaccines. These studies should include non-homologous serological testing in addition to multiple infectious challenges. The latter will provide data with which to address the issue of all-or-nothing versus leaky immunity. Also, if it is possible to validate animal models of influenza vaccination and protection with current human strains, such systems could be of considerable use in evaluating the likely efficacy of human H5 vaccines.

In this study, we have investigated the impact of the principal individual-level effects of vaccination on the general population. These effects can be quantified directly using either standard clinical trial protocols or, to some extent, simple deliberate infection experiments on isolated individuals. However, a number of secondary individual-level effects could have an impact on our findings but would be more difficult to measure directly. For example, vaccination will likely affect infectivity as well as susceptibility. Also, the elderly and the immunosuppressed may respond less well to vaccination. Although it is not clear how these variations would affect the benefits of lower-dose strategies, they would be difficult to measure because of the ethical considerations of recruiting the elderly and the immunosuppressed into vaccine trials. It may be that a single dose of pandemic vaccine can be effective in priming the immune system such that an actual infectious challenge generates a final protective response [21]. Also, novel adjuvants currently under development may be able to increase the immunogenicity of lower dose vaccinations and of cross protection between different viral clades [21]. However, here we restricted ourselves to making the best use of currently available data. Further analyses of the population level effects of both priming and novel adjuvants would need to be supported by strong empirical evidence.

The simple illustrative model, with all individuals in the population assumed to be either fully susceptible or fully immune, serves to illustrate the fundamental benefit of lower-dose strategies. Under this model, the dose-response curve *f(d)* is concave for all three candidate vaccines. Therefore, the quantity *f(d)/d* is maximized at a dose lower than the maximum, which suggests that concavity ensures that lower doses are the most efficient way to generate immune individuals. This observation motivates more detailed clinical trials of pre-pandemic vaccines. Although results are consistent for different candidate vaccines, trial data give only sparse coverage at lower doses. Also, the concept of concavity for the full model, with multiple immune states and leaky immunity, requires further theoretical investigation.

Although use of vaccine doses that give less than the maximum demonstrated protection at the individual level may present some ethical issues, they may not be as challenging as first thought. Currently, to be licensed in Europe, candidate vaccines against influenza must be able to neutralize antigens at serum concentrations of 1:40 (in HI tests) in at least 70% of individuals [22]. Draft guidelines from the US Federal Drug Administration propose that the lower bound of the 95% confidence interval for vaccine efficacy should be between 40% and 45% [23]. Typically, vaccine trials conducted for licensing purposes are designed so that the maximum dose tested just passes these hurdles. It is likely that if current seasonal influenza vaccines were tested at higher doses, they could protect a greater proportion of vaccinees without significant increases in toxicity. Therefore, to some extent, commercial pressures and the current licensing process promote the development of less-than-maximally-protective vaccines. As long as the individual-level protection of a community-optimized pandemic vaccine is clearly described, it could be argued that there is no substantive ethical difference between recommending the use of such a vaccine and recommending the use of a typical vaccine against seasonal influenza. Moreover, rationing of vital resources such as vaccines, antiviral drugs, and personal protective equipment will be inevitable in the event of a pandemic in virtually all populations, and especially so in low-resource settings. We suggest that any potential ethical dilemmas be addressed by a rationing process that is explicit, is evidence-based, and has achieved community-wide consensus.

There is a fundamental ethical difference between the prioritization of vaccine for groups within which influenza may be more transmissible due to intrinsic behavioural or immunological factors (e.g., children) and prioritization for groups that are asked to deliberately put themselves in harm's way (e.g., front-line health care workers) [24,25]. Therefore, countries with pre-pandemic vaccine stockpiles may wish to provide the most effective proven dose to health care workers while optimizing a second dose so as to reduce the overall attack rate. If the stockpile size is of the same order as the amount of antigen required to provide the maximum dose to health care workers (as is the case in the scenario we present in Table 1), and if health care workers are equally susceptible and infectious during a pandemic, then the overall efficacy of the program will be substantially reduced. However, if health care workers are more susceptible and infectious, then the overall benefit of the minimum dose policy may be greater than the values presented in Table 1. Quantifying this community-level impact of the preferential maximum strength vaccination of health care workers is challenging for a number of reasons. Although some studies have been conducted on the indirect benefits of vaccinating health care workers who work primarily with the elderly [26], these results cannot be used to derive reliable estimates of the infectivity and susceptibility of health care workers in a general setting. Further, during the main period of a pandemic, when many people would be infected in the community, a high proportion of those attending health care facilities would already be infected. Therefore, the impact of increased infectivity of health care workers may be of limited importance. We suggest that further empirical and theoretical studies are warranted in order that the likely impact of different pre-pandemic vaccination policies on health care workers and their community can be established.

Implicitly, we have assumed that the match would be good between the vaccine strain and the pandemic strain. However, it cannot be known in advance how close this match would actually be. For example, low levels of cross-reactivity have been observed between a newly emergent dominant strain of HPAI in southern China and the strains used to formulate the three vaccine candidates considered here (see Table 4 in [8]). As might be expected, our predicted benefits are reduced as the match worsens (Figure S2). Although the dominance of this new strain is probably due to high vaccine coverage of poultry in some parts of China, which is not the case elsewhere, this observation is still cause for concern. In particular, the emergence of this strain emphasizes the likely need for the constant updating of pre-pandemic vaccine stockpiles, which in practice would preclude a sufficiently large cumulative stock over time with which to provide adequate coverage for whole populations. This reinforces our argument that public health authorities must optimize the population protection derived from a limited antigen stockpile. We note that all modelling and empirical studies of pre-pandemic vaccines are, to some extent, conditional on a good match between target strains and the pandemic strain. Therefore, at the outset of a pandemic, when isolates of the circulating novel strain are available, it may be appropriate to conduct dose- and strain-specific immunogenicity trials as an integral part of the early stages of a pre-pandemic vaccination program. If such trials suggest that the vaccine is not going to be effective, it may be appropriate to stop the vaccination program.

In the event of an influenza pandemic, it seems likely that some countries will opt for transmission-limiting strategies [11] in which children and young adults are prioritized. If this is the case, we suggest that the stockpile (after provision for front-line health care workers) be large enough to offer vaccination at the lowest dose that gives an acceptable individual level of protection to all members of priority age groups. Different countries may choose different acceptable individual levels of protection, depending on their ability to manufacture or obtain antigen. If any country intends to offer widespread pre-pandemic vaccination beyond younger age groups, our results suggest that detailed transmission studies should be conducted in order to be able to predict community-level benefits, i.e., to reduce the degree of uncertainty in Figure 3. For example, if blood samples were taken from members of a large number of households before and after the annual influenza season and if the timing of symptoms were recorded, modern serological and statistical techniques would permit accurate estimates of the relative infectivity and susceptibility of different age groups. More generally, choosing pandemic vaccine doses and stockpile sizes using the approaches described here will help to ensure that entire communities receive optimal benefits from limited resources.

## Supporting Information

Alternative Language Abstract S1 Translation of the Abstract into Simplified Chinese by JTW and GML (24 KB DOC)

Alternative Language Abstract S2 Translation of the Abstract into Traditional Chinese by JTW and GML (26 KB DOC)

Figure S1 Sensitivity of Predicted Reduction in Attack Rate to the Shape of the Relation between HI Levels and Probability of Infection (84 KB DOC)

Figure S2 Sensitivity of Results from the Homogeneous Model to Closeness of Match between Vaccine Strain and Pandemic Strain (100 KB DOC)

Table S1 Rates of Infection of Volunteers from Deliberate and Natural Infection with Three Strains of Influenza (36 KB DOC)

## Abbreviations

HI: haemagglutinin inhibition
HPAI: highly pathogenic avian influenza
