# Supplementary material for: Optimizing the Dose of Pre-Pandemic Influenza Vaccines to Reduce the Infection Attack Rate
Source: PLoS Med. 2007 Jun 19;4(6):e218. doi: 10.1371/journal.pmed.0040218 (PMC1892041; doi:10.1371/journal.pmed.0040218)
Supplement: Supplementary file 1 [file pmed.0040218.sd001.doc]

近年广泛传播的禽流感有可能会引发流感大流行。有见及此，多个国家正考虑预先储备适用於人类的禽流感疫苗。禽流感抗原的数量将会是决定疫苗储备量的主要因素。

调低疫苗的抗原剂量虽会减弱疫苗的效力，但疫苗的数量能得以增加从而提高疫苗注射的覆盖率。这种做法或许能减低感染流感的人数。我们运用数学模型模拟人类对不同抗原剂量的反应和流感的传播，从而预测疫苗抗原剂量如何影响感染人数。我们发现调低抗原剂量来增加注射疫苗的人数能有效地减低感染率。这结论和迄今已进行临床测试的三种禽流感疫苗的数据均吻合。我们利用过往的疫苗数据估计感染率的降幅。以Lin et al 的疫苗和美国人口(三亿)为例，如果将二千万剂10μg的疫苗(美国为应付感染大流行而定出的剂数)变为一亿六千万剂1.25μg的疫苗，感染率将由67.7%降至58.7%。人类对疫苗的反应难以仔细确定，我们依据这方面的研究资料对感染率的降幅作出最保守的估计。如果疫苗的储备量只足够用於小童(一群较易感染和传染流感的人口) ，即使小童的感染度和传染度高至其他人口的两倍，感染率的降幅将高於以上的估计。

各国设定疫苗储备量和剂量时，除了考虑每剂疫苗的效力外，还应考虑疫苗注射对整体人口感染率的影响。这项研究指出调低疫苗的抗原剂量来提高注射覆盖率和减低感染率的可能性。
