# Supplementary material for: Optimizing the Dose of Pre-Pandemic Influenza Vaccines to Reduce the Infection Attack Rate
Source: PLoS Med. 2007 Jun 19;4(6):e218. doi: 10.1371/journal.pmed.0040218 (PMC1892041; doi:10.1371/journal.pmed.0040218)
Supplement: Supplementary file 2 [file pmed.0040218.sd002.doc]

近年廣泛傳播的禽流感有可能會引發流感大流行。有見及此，多個國家正考慮預先儲備適用於人類的禽流感疫苗。禽流感抗原的數量將會是决定疫苗儲備量的主要因素。

調低疫苗的抗原劑量雖會減弱疫苗的效力，但疫苗的數量能得以增加從而提高疫苗注射的覆蓋率。這種做法或許能減低感染流感的人數。我們運用數學模型模擬人類對不同抗原劑量的反應和流感的傳播，從而預測疫苗抗原劑量如何影響感染人數。我們發現調低抗原劑量來增加注射疫苗的人數能有效地減低感染率。這結論和迄今已進行臨床測試的三種禽流感疫苗的數據均吻合。我們利用過往的疫苗數據估計感染率的降幅。以Lin et al 的疫苗和美國人口(三億)為例，如果將二千萬劑10μg的疫苗(美國為應付感染大流行而定出的劑數)變為一億六千萬劑1.25μg的疫苗，感染率將由67.7%降至58.7%。人類對疫苗的反應難以仔細確定，我們依據這方面的研究資料對感染率的降幅作出最保守的估計。如果疫苗的儲備量只足夠用於小童(一群較易感染和傳染流感的人口) ，即使小童的感染度和傳染度高至其他人口的兩倍，感染率的降幅將高於以上的估計。

各國設定疫苗儲備量和劑量時，除了考慮每劑疫苗的效力外，還應考慮疫苗注射對整體人口感染率的影響。這項研究指出調低疫苗的抗原劑量來提高注射覆蓋率和減低感染率的可能性。
