# Supplementary material for: Optimizing the Dose of Pre-Pandemic Influenza Vaccines to Reduce the Infection Attack Rate
Source: PLoS Med. 2007 Jun 19;4(6):e218. doi: 10.1371/journal.pmed.0040218 (PMC1892041; doi:10.1371/journal.pmed.0040218)
Supplement: Supplementary file 3 [file pmed.0040218.sg001.doc]

**Supporting Figure S1.** Sensitivity of predicted reduction in attack rate to the shape of the relation between HI levels and probability of infection. In **A,** the squares show the point estimate $p_{\text{val}}$ for the probability of being infected conditional on being having HI titre $h_{\text{val}}$ during the Pre-1968 A2 Salisbury trial [1]. The vertical bars show independent 95% binomial confidence intervals for each estimated probability. Note that Figure 6 from Ref [1] was used for the probability and Figure 5 for the sample size within each HI class. The solid line shows the maximum likelihood estimate for the continuous function $p_{\text{inf}}(h_{\text{val}};A,\gamma)=ae^{-h_{\text{val}}\gamma}$. The dashed lines are univariate 95% prediction intervals for $\gamma$ (note that only the shape of $p(h;a,\gamma)$ is relevant for the mapping from HI to protection). We also considered the functional form $p_{\text{inf}}(h_{\text{val}};A,\gamma)=ae^{-h_{\text{val}}\min\{(\gamma-d),0\}}$. However, the addition of the offset parameter $d$ did not improve the model fit. **B** shows the sensitivity of the predicted reduction in attack rate for the Lin vaccine [2] to different sizes of stockpile (x-axis) and different values of the shape parameter $\gamma$ which lie within the 95% confidence region. The dashed line shows the maximum likelihood value of $\gamma$. The white cross marks the scenario used for sensitivity analyses in Table 1, namely sufficient vaccine for 20 million doses in a population of 300 million at the maximum dose. The reduction in attack rate for this scenario under the statistical model is 5.9%.

**References**

1. Hobson D, Curry RL, Beare AS, Ward-Gardner A (1972) The role of serum haemagglutination-inhibiting antibody in protection against challenge infection with influenza A2 and B viruses. J Hyg (Lond) 70: 767-777.

2. Lin J, Zhang J, Dong X, Fang H, Chen J, et al. (2006) Safety and immunogenicity of an inactivated adjuvanted whole-virion influenza A (H5N1) vaccine: a phase I randomised controlled trial. Lancet 368: 991-997.
