# Supplementary material for: Optimizing the Dose of Pre-Pandemic Influenza Vaccines to Reduce the Infection Attack Rate
Source: PLoS Med. 2007 Jun 19;4(6):e218. doi: 10.1371/journal.pmed.0040218 (PMC1892041; doi:10.1371/journal.pmed.0040218)
Supplement: Supplementary file 4 [file pmed.0040218.sg002.doc]

**Supporting Figure S2.** Sensitivity of results from the homogeneous model to closeness of match between vaccine strain and pandemic strain. Charts show how predicted reductions in attack rate (chart color, see legend) for the optimal policy compared with the use of maximum dose policy are affected by a reduced match of the pandemic strain with the pre-pandemic vaccine (y-axes) for different stockpile sizes (x-axes): **A** Treanor et al [1], **B** Bresson et al [2] and **C** Lin et al [3]. To describe reduced protection from distinct strains, we assume that that the protection level in each immune class $z_{i}\to mz_{i}$ (see main text), where $m$ is the closeness of the antigenic match (y-axes).

**References**

1. Treanor JJ, Campbell JD, Zangwill KM, Rowe T, Wolff M (2006) Safety and immunogenicity of an inactivated subvirion influenza A (H5N1) vaccine. N Engl J Med 354: 1343-1351.

2. Bresson JL, Perronne C, Launay O, Gerdil C, Saville M, et al. (2006) Safety and immunogenicity of an inactivated split-virion influenza A/Vietnam/1194/2004 (H5N1) vaccine: phase I randomised trial. Lancet 367: 1657-1664.

3. Lin J, Zhang J, Dong X, Fang H, Chen J, et al. (2006) Safety and immunogenicity of an inactivated adjuvanted whole-virion influenza A (H5N1) vaccine: a phase I randomised controlled trial. Lancet 368: 991-997.
