# Supplementary material for: Optimizing the Dose of Pre-Pandemic Influenza Vaccines to Reduce the Infection Attack Rate
Source: PLoS Med. 2007 Jun 19;4(6):e218. doi: 10.1371/journal.pmed.0040218 (PMC1892041; doi:10.1371/journal.pmed.0040218)
Supplement: Supplementary file 5 [file pmed.0040218.st001.doc]

**Supporting Table S1.** Rates of infection of volunteers from deliberate^a^ and natural^b^ infection with three strains of influenza [1].

| HI Titer | HK Salisbury trial^a^ | Pre-1968 A2 Salisbury trial^a^ | HK field trial^b^ |
| --- | --- | --- | --- |
| <6 | 74.3% | 67.7% | 88.7% |
| 6 | 82.6%^c^ | 90.9%^c^ | 49.8% |
| 12 | 76.2% | 66.1% | 34.5% |
| 24 | 41.9% | 53.3% | 29.5% |
| 48 | 17.4% | 31.2% | 19.9% |
| 96 | 8.78% | 22.4% | 1.88% |
| 192 | 4.23% | 0.78% | 1.88% |
| 384 | 0.00% | 0.00% | 0.00% |

^c^ We use the class with the highest attack rate as the baseline for susceptibility for all results presented in the main text – effectively imposing the constraint that the relation between HI titre and protection is a monotonically decreasing function. The apparent inconsistency that individuals with HI titres of 1:6 had greater infection rates than those with HI less than 1:6 is likely due to the presence of pre-existing immunity in the <1:6 group which was not measured by the HI assay {Hobson, 1972 #147}. Such pre-existing immunity is unlikely to occur with a pandemic strain. Even so, the choice of baseline does not qualitatively affect our results because vaccine trial data are only available for HI titres of 10 or greater. However, the absolute degree of protection implied by a given HI titre is dependent on which class is chosen as a baseline for susceptibility. In Supporting Figure S2, we investigate this issue further using a parametric model of the relation between HI titre and reduced susceptibility.

**Reference**

1. Hobson D, Curry RL, Beare AS, Ward-Gardner A (1972) The role of serum haemagglutination-inhibiting antibody in protection against challenge infection with influenza A2 and B viruses. J Hyg (Lond) 70: 767-777.
